# Canted antiferromagnetic order in EuZn_2_As_2_ single crystals

**DOI:** 10.1038/s41598-022-19026-6

**Published:** 2022-08-30

**Authors:** Zbigniew Bukowski, Damian Rybicki, Michał Babij, Janusz Przewoźnik, Łukasz Gondek, Jan Żukrowski, Czesław Kapusta

**Affiliations:** 1 Institute of Low Temperature and Structure Research, Polish Academy of Sciences, ul. Okólna 2, 50-422 Wrocław, Poland; 2 Faculty of Physics and Applied Computer Science, AGH University of Science and Technology, Av. A. Mickiewicza 30, 30-059 Kraków, Poland; 3 Academic Centre for Materials and Nanotechnology, AGH University of Science and Technology, Av. A. Mickiewicza 30, 30-059 Kraków, Poland

**Keywords:** Magnetic properties and materials, Electronic properties and materials

## Abstract

Compounds containing Eu show a vast range of unique physical properties due to the interplay of electronic and magnetic properties, which can lead to a nontrivial electronic topology combined with magnetic order. We report on the growth of trigonal ($P\overline{3 }m1$ space group) EuZn_2_As_2_ single crystals and on the studies of their structural, electronic and magnetic properties. A range of experimental techniques was applied including X-ray diffraction, electron microscopy, magnetic susceptibility, magnetization, heat capacity and Mössbauer spectroscopy in the study. We found that Eu has solely a 2+ valence state and its magnetic moments below *T*_*N*_ = 19.2 K form a canted antiferromagnetic structure, tilted from the basal plane.

## Introduction

Rare earth based compounds show multitude of interesting and unique magnetic and electronic properties. Materials containing Eu are not an exception with this regard with their properties being a result of interactions between the localized 4*f* electrons and conduction electrons via the RKKY coupling^1,2^. Eu is particularly interesting, since it appears in two valence states, which cause very different physical properties. In intermetallic compounds, the magnetic Eu^2+^ configuration (with *4f*^7^, *L* = 0, *S* = *J* = 7/2) is more often observed than the non-magnetic Eu^3+^ one (4*f*^6^, *L* = *S* = 3, *J* = 0). In some materials an intermediate valence state is also possible, e.g. in EuZnSb_2_^3^ or EuNi_2_P_2_^4^. These two compounds are a good example of a wide range of different physical properties as the first one is an antiferromagnetic semimetal with Dirac states, and the second one shows a heavy fermion behavior and the Kondo effect. Combination of magnetic and electronic properties can lead to effects like colossal magnetoresistance^5^, but the fact that many Eu compounds show nontrivial electronic topology, which is combined with magnetic order, is even more interesting. The examples of such materials are EuCd_2_As_2_^6,7^ and EuIn_2_As_2_^8^, which are a topological semimetal and an insulator, respectively. Both compounds have hexagonal crystal structure, order antiferromagnetically and have similar ordering temperature as EuZn_2_As_2_ studied by us, which is expected to be a topological semimetal^9^. We undertook the study of EuZn_2_As_2_ in order to determine its magnetic structure since such knowledge is crucial for the elucidation of possible nontrivial surface states of magnetic topological materials.

Here, we report on the growth of large, high quality single crystals of EuZn_2_As_2_ and the studies of their structural, electronic and magnetic properties by means of X-ray diffraction, electron microscopy, magnetization and AC susceptibility, heat capacity, and ^151^Eu Mössbauer spectroscopy.

## Experimental

Single crystals of EuZn_2_As_2_ were grown by the high-temperature solution technique using tin as a flux. Starting materials: europium (3N), zinc (4N), arsenic (4N) and tin (4N) pieces weighed in the atomic ratio Eu: Zn: As: Sn = 1: 2: 2: 20, were placed into an alumina crucible and sealed under vacuum in a silica glass ampule. The ampoule was heated in a resistance furnace to 1050 °C within 10 h, then kept at this temperature for 20 h and finally cooled slowly (2–3 °C/h) to 600 °C. The single crystals were separated from the flux using a centrifuge. Samples quality was checked by scanning electron microscopy (SEM) using a JEOL 5900LV microscope equipped with an energy-dispersive X-ray spectrometer (EDS).

Powder X-ray diffraction (XRD) measurements were made by a Panalytical Empyrean diffractometer. For the low temperature XRD studies an Oxford Instruments PheniX closed-cycle helium refrigerator was used (14–300 K). During the low-temperature measurements the sample position was corrected against thermal displacement of the sample stage. The stabilization of temperature was better than 0.1 K. The XRD patterns were refined using the Rietveld-type package FullProf^10^.

The DC mass susceptibility/magnetization were measured in the temperature range of 2–300 K in magnetic fields up to 9 T using the vibrating sample magnetometer (VSM) option of a Quantum Design Physical Property Measurement System (PPMS-9). The AC susceptibility was measured in the temperature range of 2–50 K in magnetic fields up to 9 T using the AC measurement system (ACMS) option in a PPMS-9.

The heat capacity measurements were carried out by a two-tau relaxation method with the heat capacity option of a PPMS-9 during heating in the temperature range of 1.85–295 K. The single crystal samples for heat capacity measurements were attached and thermally coupled to the addenda with Apiezon N grease. A background signal from addenda and grease was recorded versus temperature.

^151^Eu Mössbauer spectra were recorded at 300 K and at 4.2 K with a conventional constant acceleration spectrometer using the ^151^Sm(SmF_3_) source. The 21.5 keV *γ*-rays were detected with a NaI(Tl) scintillation detector. The absorber surface density was approx. 60 mg/cm^2^. The spectra were analyzed by means of a least-squares fitting procedure. The absorption line positions and relative intensities were calculated by numerical diagonalization of the full hyperfine interactions Hamiltonian. The fitted hyperfine parameters are *δ*, *ε*, *B* and *θ*, where *δ* stands for the isomer shift given relative to the ^151^Sm(SmF_3_) source at room temperature. *ε* = ¼(*c*/*E*_*γ*_)*eQ*_*g*_*V*_*zz*_ is the quadrupole coupling parameter for Eu, assuming that the electric field gradient (EFG) is axially symmetric. Symbol *c* stands for the speed of light in vacuum, *E*_*γ*_—Mössbauer photon energy, *e—*elementary charge value, *Q*_*g*_—nuclear electric quadrupole moment in the ground state of ^151^Eu. *B* denotes the hyperfine magnetic field at the nucleus and *θ* stands for the angle between the principle component (*V*_*zz*_) axis of the EFG and the hyperfine magnetic field at the Eu nucleus.

## Results and discussion

### Structural properties

**Figure 1:**
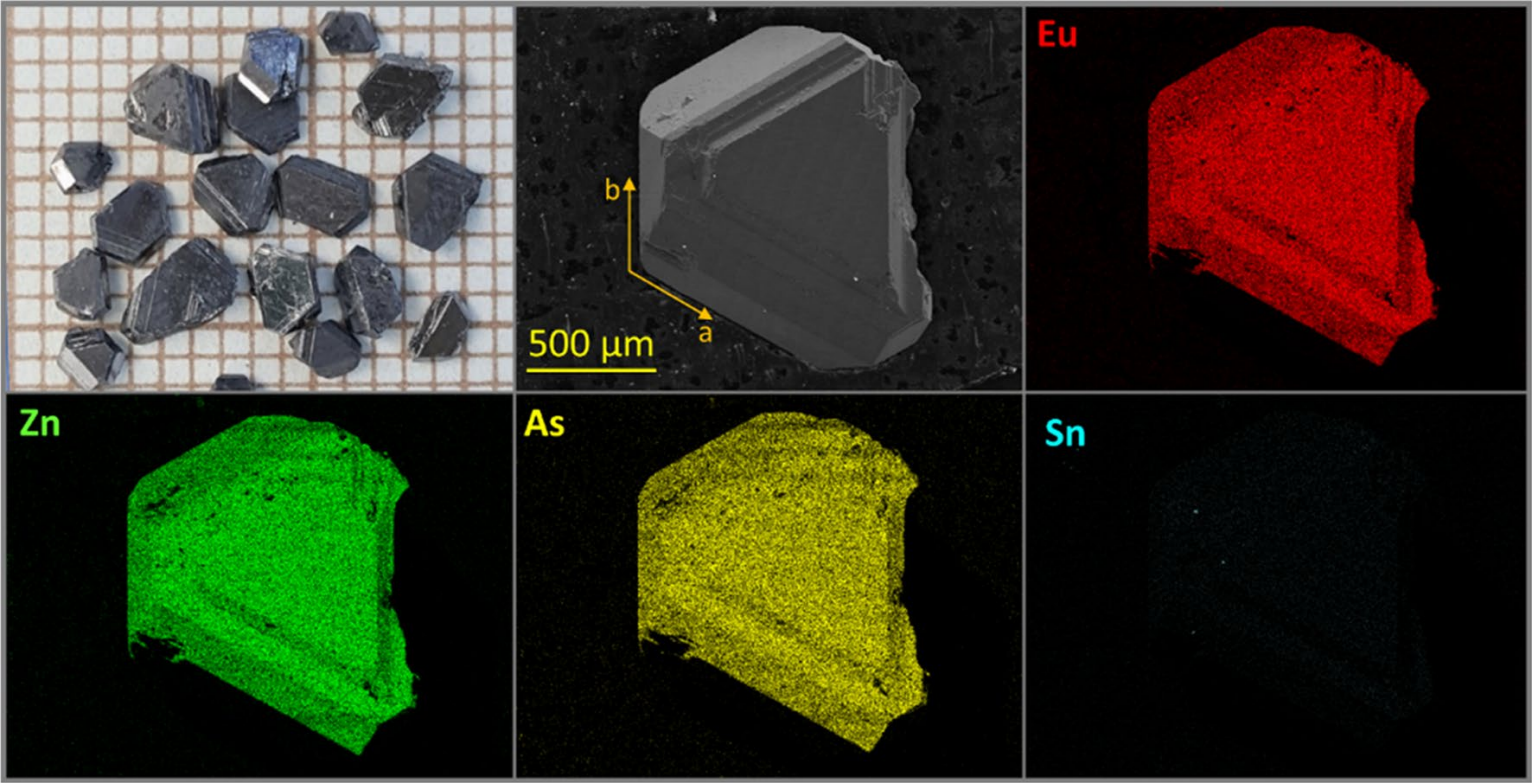
Single crystals of EuZn_2_As_2_ grown from Sn flux together with SEM image and X-ray microanalysis data of elemental distributions.

The obtained single crystals were up to a few mm in size and have well developed shapes corresponding to the crystal symmetry, according to Fig. 1. The planes with the largest surface are parallel to the [001] family of planes, as checked by X-ray diffraction. Also [100] and [010] directions (with angle of 120°) can be easily distinguished by eye and confirmed by XRD. The crystals exhibit good homogeneity, as apparent from Fig. 1. The elemental distribution over the chosen piece of specimen shows only traces of remaining Sn from the flux on the crystal surface. The results of the X-ray microanalysis confirmed that the ratio of Eu:Zn:As is approximately 1:2:2, indicating that the composition of the sample is stoichiometric EuZn_2_As_2_ within the measurement uncertainty of 5%.

**Figure 2:**
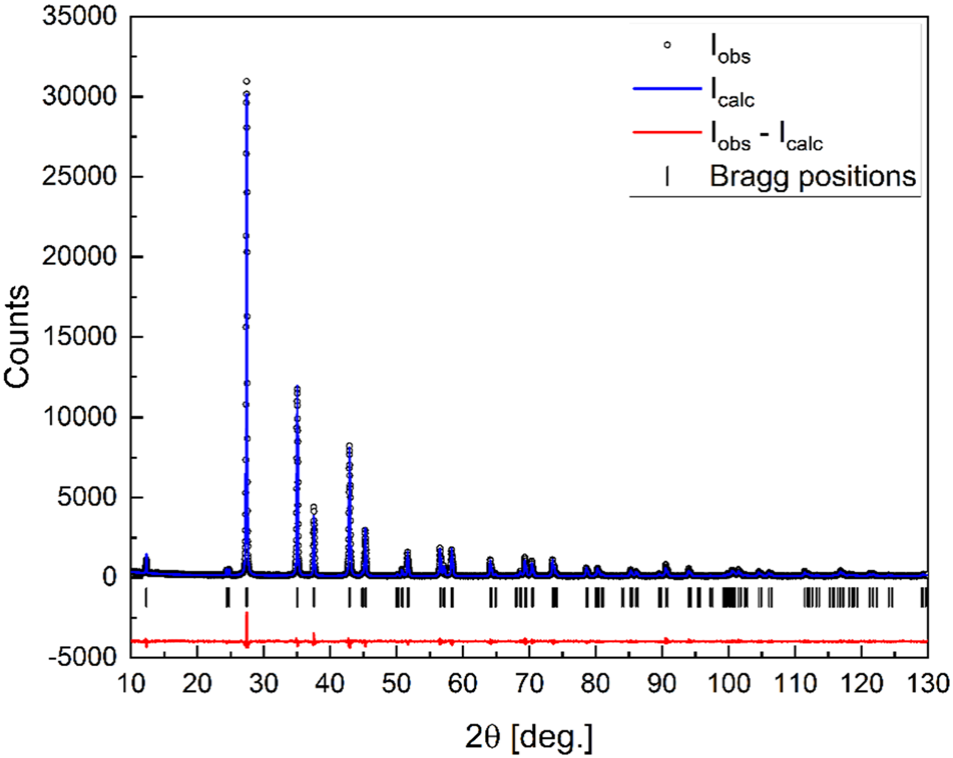
Refined X-ray diffraction pattern of EuZn_2_As_2_.

**Figure 3:**
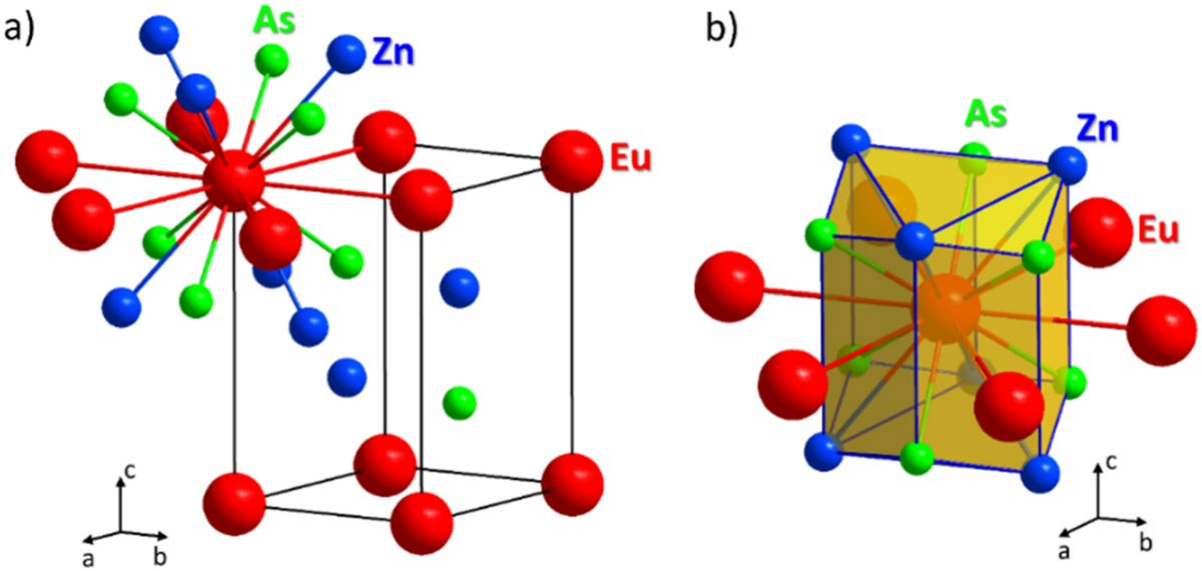
Crystal structure of EuZn_2_As_2_, (**a**) with coordination of Eu atom, (**b**) showing that the *c*-axis is a threefold rotation axis, leading to an axially symmetric electric field gradient at the Eu site. Figure was created using Diamond v. 4.6.3, https://www.crystalimpact.com/diamond/.

The XRD pattern of EuZn_2_As_2_ at 300 K can be entirely indexed by the trigonal $P\overline{3 }m1$ space group (No. 164). No spurious phases were observed revealing excellent sample quality, as can be seen in Fig. 2. The corresponding lattice parameters are: *a* = 4.2127(1) Å and *c* = 7.1837(2) Å. The refined parameters are consistent with previous findings on powder samples^11^. The crystal structure and coordination of the Eu atoms are shown in Fig. 3. The existence of a threefold rotation axis parallel to the [001] direction can be easily noticed by analysis of the triangular Zn and As distribution with respect to the [001] direction (Fig. 3b). Another interesting aspect of this crystal is layered structure, where Zn and As form honeycomb layers, separated by Eu planes. Therefore, the structure promotes complex and strongly anisotropic magnetic properties, especially in the case of antiferromagnetic exchange in the basal *ab* plane.

**Figure 4:**
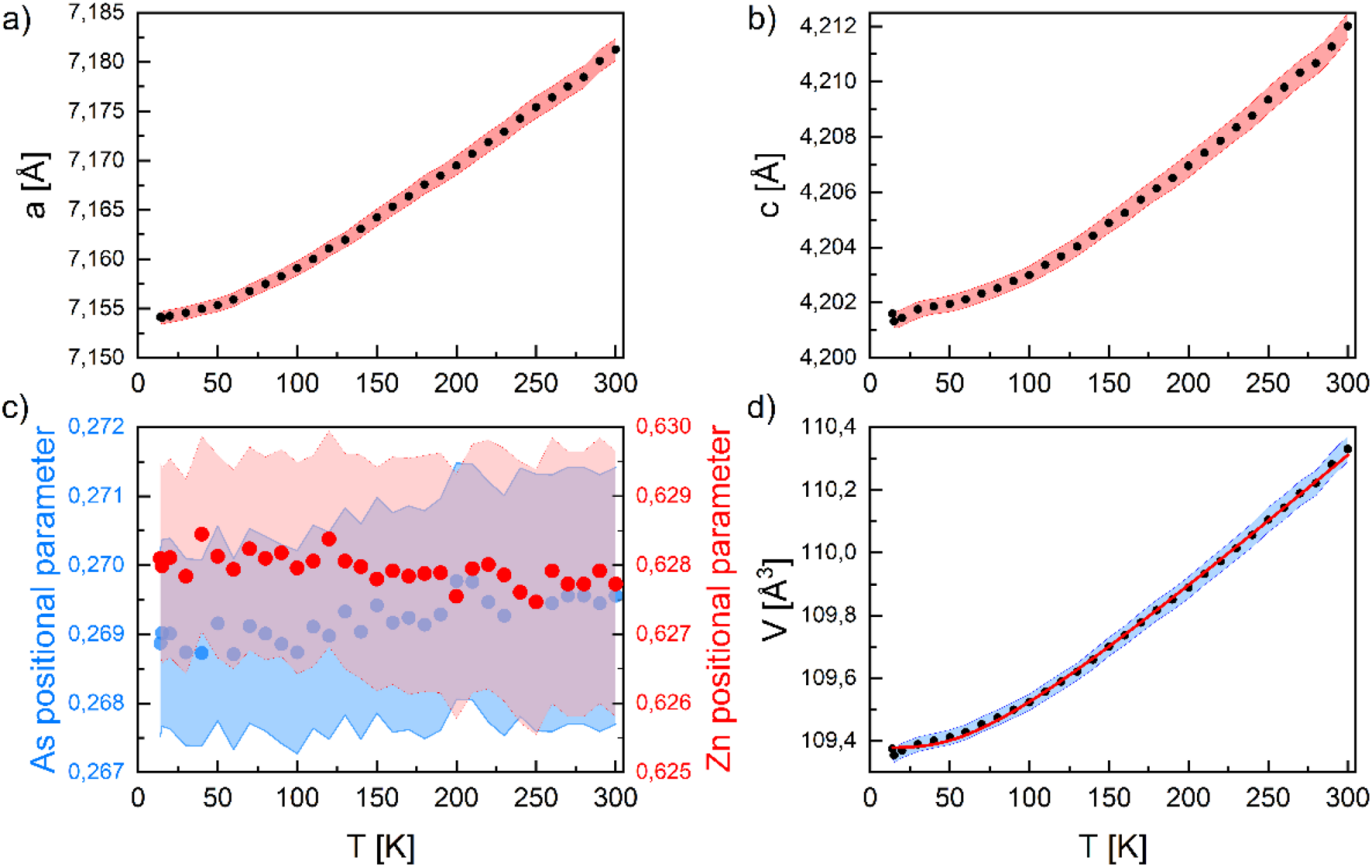
Temperature dependences of lattice parameters: (**a**) *a* lattice parameter; (**b**) *c* lattice parameter; (**c**) As and Zn positional parameters; (**d**) unit cell volume (the red curve is the Debye function fit discussed in text). Colored areas represent experimental uncertainties. Figure was created using Origin v. 2020, https://www.originlab.com/.

At low temperatures (down to 14 K) the crystal structure of EuZn_2_As_2_ remains unchanged. The only changes observed in XRD patterns are due to a thermal expansion of the unit cell, as depicted in Fig. 4a,b. In the entire experimental range no significantly anomalous behavior can be noticed. The only small deviation of the *a* lattice parameter, and consequently the unit cell volume, can be noticed at low temperature at 15 K. Interestingly enough the *c* lattice parameter does not show contraction at this temperature. Positional parameters of Zn and As do not change within uncertainty levels over the entire temperature range, as can be inferred from the Fig. 4c.

Temperature changes of the unit cell volume, presented in Fig. 4d, can be roughly described using the Debye formula^12^:

1 $$ V = V_{0} + I_{C} \frac{{T^{4} }}{{\theta_{D}^{3} }}\int_{0}^{{\frac{{\theta_{D} }}{T}}} {\frac{{x^{3} }}{{e^{x} - 1}}dx} $$

where ${V}_{0}$ is the unit cell volume at 0 K, ${I}_{C}$ is the coefficient involving the Grüneisen and compressibility parameters and ${\theta }_{D}$ is the Debye temperature. The ${I}_{C}$ coefficient is a slope of the linear part of the $V\left(T\right)$ dependence. The refined parameters are as follows: ${V}_{0}$ = 109.38(1) Å^3^, ${I}_{C}$ = 0.0132(1) Å^3^/K and ${\theta }_{D}$ = 261(8) K.

### Magnetic properties

**Figure 5:**
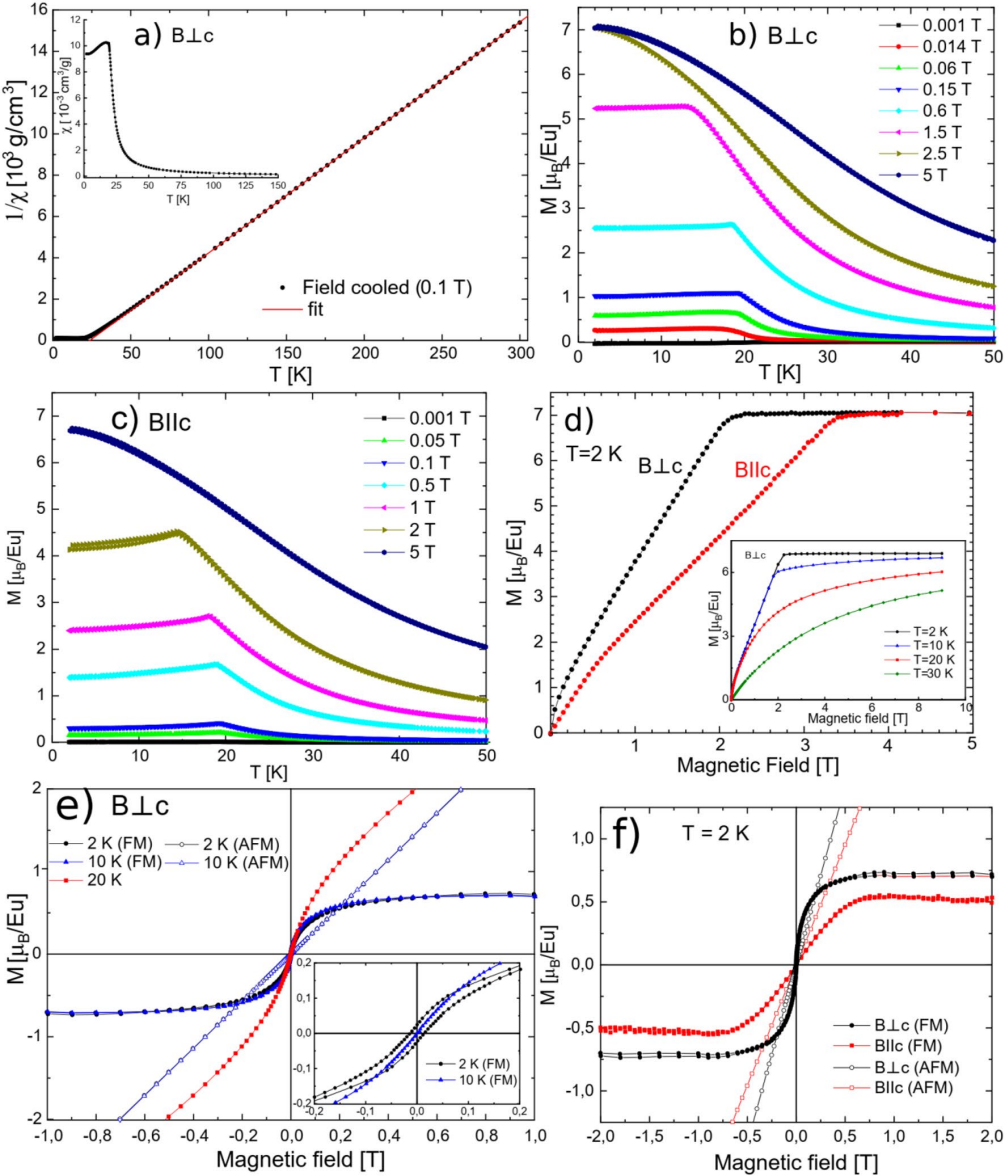
(**a**) The temperature dependence of the inverse DC magnetic susceptibility 1/*χ* with the Curie–Weiss law fit (between 110 and 300 K) and *χ*(*T*) in the inset, (**b**) and (**c**) the temperature and field dependences of the magnetization *M* for the field perpendicular (*B*⊥*c*) and parallel (*B*||*c*) to the *c*-axis, respectively, (**d**) field dependence of *M* measured at 2 K for *B*⊥*c* and *B*||*c*, the inset shows measurements at different temperatures for *B*⊥*c*, (**e**) results of decomposition of magnetization into ferromagnetic (FM) and antiferromagnetic (AFM) components (for 2 K and 10 K), the inset shows a zoomed view of FM components, (**f**) FM and AFM components for *B*⊥*c* and *B*||*c* at 2 K.

Results of measurements of magnetic properties are presented in Fig. 5. Inset of panel a) shows the temperature dependence of the low field magnetic susceptibility *χ*, for the field perpendicular to the *c*-axis (*B*⊥*c*). It increases with decreasing temperature until the kink at temperature *T*_*N*_ = 19.2 K is reached, where it first saturates and then slightly decreases, indicating a transition to antiferromagnetic (AFM) state. This temperature is close to that reported recently for polycrystalline^13^ and single crystalline samples^14^. Panel (a) also presents the temperature dependence of the reciprocal magnetic susceptibility 1/*χ*, showing a linear behavior above *T*_*N*_, together with the fit of the Curie–Weiss dependence: $\chi \left(T\right)={\chi }_{0}+\frac{C}{\left(T-{\theta }_{p}\right)}$. The values of ${\chi }_{0}=-\left(4.8\pm 0.4\right){10}^{-7}\frac{{\mathrm{cm}}^{3}}{\mathrm{g}}$, $C=\left(18.05\pm 0.02\right){10}^{-3}\frac{{\mathrm{cm}}^{3}\mathrm{K}}{\mathrm{g}}$ and ${\theta }_{\mathrm{p}}=23.8\pm 0.1 K$ were obtained. From the Curie constant *C* we calculated the effective magnetic moment ${\mu }_{eff}=7.90\pm 0.01{ \mu }_{B}$ per Eu atom (assuming a sole Eu origin of magnetism), which is very close to the theoretical value for paramagnetic Eu^2+^ ($7.94{ \mu }_{B}$), of the 4*f*^7^ electronic configuration. A positive value of the paramagnetic Curie temperature ${\theta }_{p}$ indicates dominating ferromagnetic correlations between the Eu magnetic moments in the paramagnetic state.

The temperature dependences of magnetization at various magnetic fields are presented in panels (b) and (c). One can notice that for both orientations of magnetic field with respect to the *c*-axis *T*_*N*_ decreases with increasing field as is expected for an antiferromagnet. At sufficiently high field (2.5 T for *B*⊥*c* and at 5 T for *B*||*c*), the kink corresponding to *T*_*N*_ vanishes.

**Figure 6:**
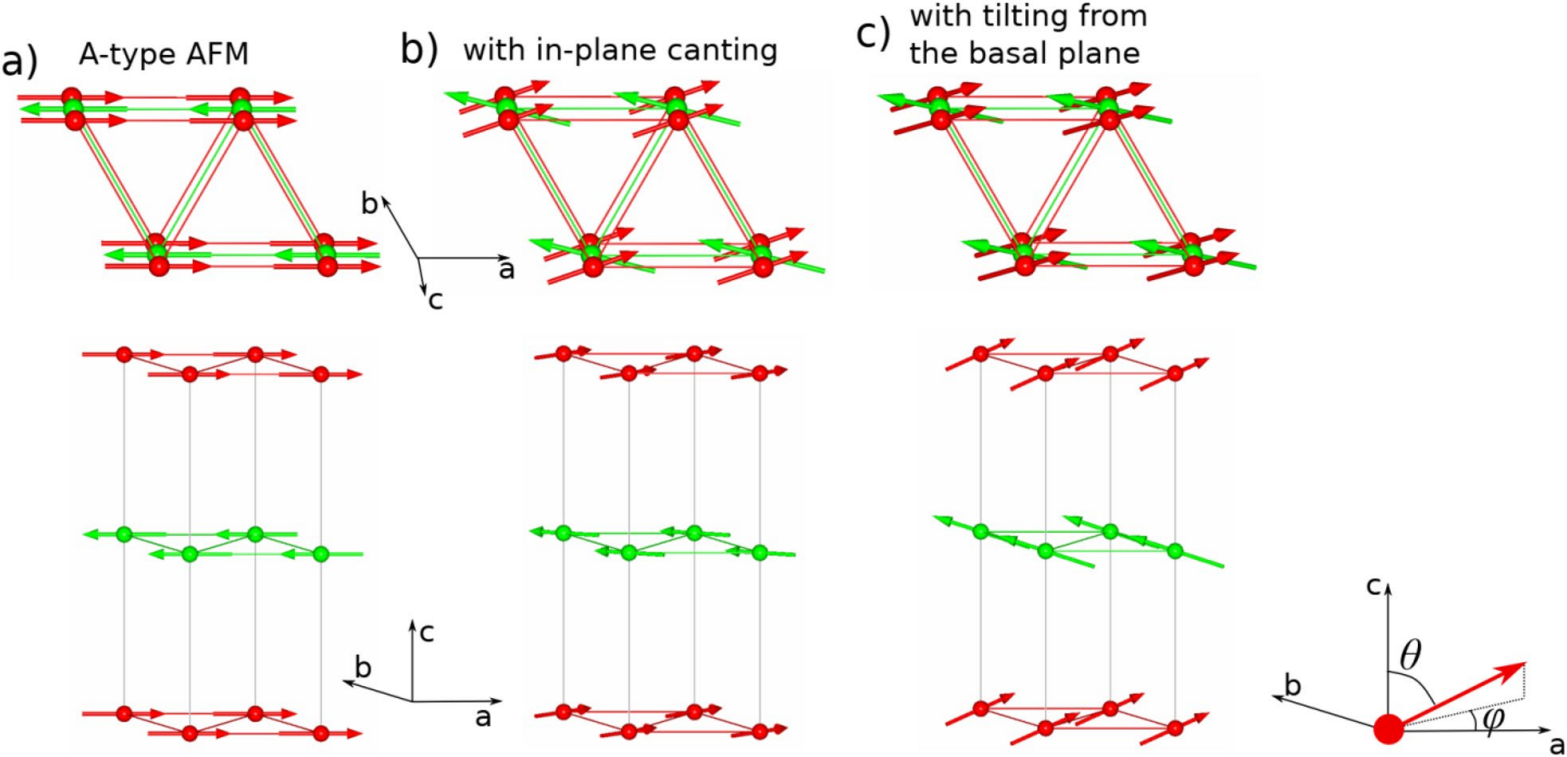
Schemes of antiferromagnetic order considered: (**a**) A-type, (**b**) with in-plane canting, (**c**) with tilting from the basal plane. Figure was created using FullProf Studio v. 2.0 https://www.ill.eu/sites/fullprof/ and Inkscape v. 1.0.1 https://inkscape.org/.

Panel (d) presents field dependences of magnetization for both orientations of the magnetic field with respect to the crystallographic axes, measured at 2 K, i.e. much below *T*_*N*_. Both curves reach saturation, which occurs for *B*⊥*c* at 2.2 T and for *B*||*c* at 3.4 T. This suggests that the *c*-axis direction is magnetically harder than that in the plane perpendicular to it. The saturation magnetization values correspond to $7{ \mu }_{B}$ per Eu atom, which is expected for Eu^2+^ ions in the magnetically ordered state. The *M*(*H*) dependences in panel d, show clear nonlinearities at low field for both orientations. In the recent study on EuZn_2_As_2_^14^ linear *M*(*H*) dependences were observed in the whole field range below saturation. This difference with respect to our results might be caused by different magnetic structure. In ref^14^ the A-type antiferromagnetic magnetic structure was proposed (Fig. 6a). For EuSn_2_As_2_, which also has a trigonal structure, two types of magnetic structures were proposed by different research groups: the A-type and a canted one (Fig. 6b)^15,16^. Pakhira et al.^16^ even reported very different temperature dependences of magnetic susceptibility for single crystals grown at different conditions.

Both of our *M*(*H*) curves (*B*⊥*c* and *B*||*c*) measured are found to correspond to hard magnetization directions of an antiferromagnet as we do not observe features characteristic for an easy magnetization direction (e.g. spin flop)^17^. Although Blawat et al.^14^ claim an A-type antiferromagnetic structure, no spin flop was observed for *B*⊥*c* either. In the inset of Fig. 5d results for *B*⊥*c* orientation at different temperatures are shown. The *M*(*H*) dependence at 10 K is almost the same as at 2 K with somewhat smaller saturation magnetization value. The result at 20 K (i.e. 1 K above *T*_*N*_) still shows similarity to that at 10 K, but magnetization does not reach saturation up to 9 T. At 30 K, in the paramagnetic region, it still shows a nonlinear dependence. Similar non-linear *M*(*H*) dependence was observed in EuSn_2_As_2_ single crystals, but only for *B*⊥*c* orientation, which was attributed to a canted antiferromagnetic structure with a ferromagnetic component in the plane perpendicular to the *c*-axis, as shown in Fig. 6b^15^. In our case such non-linear behavior is observed for both orientations. This suggests that the ferromagnetic component departs from the basal plane (Fig. 6c).

We approximated the antiferromagnetic component with a straight line (fitted at high fields) and subtracted it from the total *M*(*H*), which allowed us to obtain the ferromagnetic component^18^. This procedure was carried out for measurements at 2 K and 10 K and its results for *B*⊥*c* orientation are shown in Fig. 5e. The saturation magnetization value and the field at which saturation of the ferromagnetic component is reached are almost the same at both temperatures. In the inset to the Fig. 5e a zoomed view on the ferromagnetic component at 2 K and 10 K are shown. At 2 K this component shows a non-zero, but very small (1.2 mT) coercive field, which practically vanishes at 10 K. Figure 5e shows also antiferromagnetic components and the *M*(*H*) dependence at 20 K for a comparison. In Fig. 5f we compare the derived ferromagnetic and antiferromagnetic components for *B*⊥*c* and *B*||*c* orientations measured at 2 K. The saturation magnetization value of the ferromagnetic component is higher for *B*⊥*c* orientation (0.72 ${\mu }_{B}$ per Eu atom) than for *B*||*c* (0.52 ${\mu }_{B}$ per Eu atom). Unlike the *B*⊥*c,* for *B*||*c* no coercivity is observed. For *B*⊥*c* the magnetization of the ferromagnetic component initially increases rapidly, but the increase slows down at higher fields, and then a saturation is reached. This behavior suggests that the direction perpendicular to the *c*-axis is an intermediate magnetizing direction for the ferromagnetic component, although being close to an easy direction. On the contrary, the *c*-axis magnetization curve is found to correspond to the hard magnetizing direction here, analogously to typical magnetizing curves of ferromagnets^17^. It is worth noting that in our case the Eu sites have six equivalent directions in the plane perpendicular to the *c*-axis and, thus, easier magnetizing in the plane than along the *c*-axis, is expected. Taking the above into account we conclude that our EuZn_2_As_2_ single crystals exhibit a canted antiferromagnetic structure with magnetic moments tilted from the basal plane (Fig. 6c). This discrepancy with respect to the A-type order proposed by Blawat et al.^14^ is consistent with difference in coupling between Eu magnetic moments, as indicated by a 50% higher paramagnetic Curie temperature in our case. Also the unit cell volume is larger (by 0.3%) in our case, which altogether can explain the different magnetic structures. As we mentioned above, both types of magnetic structures are reported for EuSn_2_As_2_ by different groups^15,16^.

**Figure 7:**
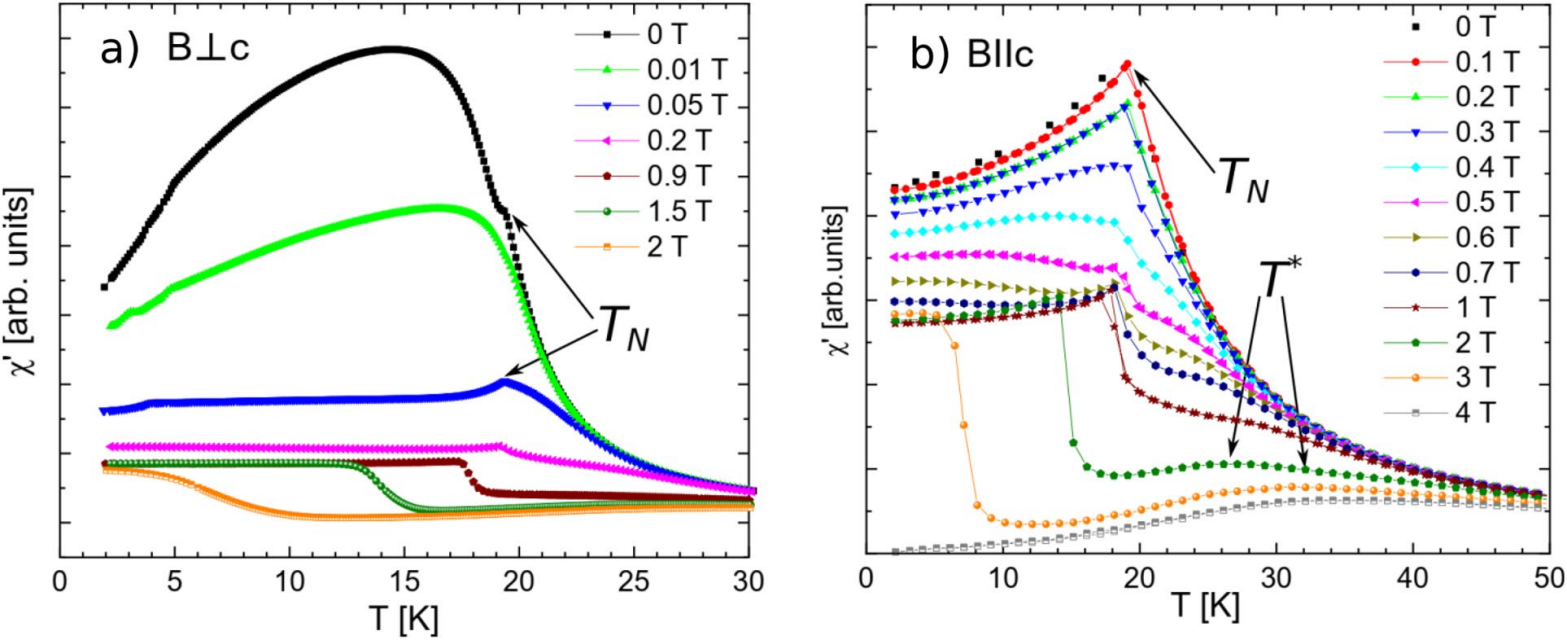
(**a**) and (**b**) The temperature and the field dependences of the real component of AC magnetic susceptibility *χ′* for the static field perpendicular and parallel to the *c*-axis, respectively.

Panels (a) and (b) of Fig. 7 present temperature dependences of the real part of the AC magnetic susceptibility *χ′* at different applied field for both orientations with the AC magnetic field parallel to the static field. The *χ′* generally increases with lowering temperature until reaching a maximum corresponding to magnetic ordering and then it decreases. For *B*⊥*c* at zero field a kink is observed at *T*_*N*_ and *χ′* further increases and then smoothly decreases with lowering temperature. At 0.01 T only a weak anomaly occurs at *T*_*N*_, which is clearly visible in the derivative (not shown). With increasing static field the kink is again well visible and shifts to lower temperature, eventually vanishing at high fields (around 2.2 T and 3.4 T for *B*⊥*c* and *B*||*c*, respectively), where antiferromagnetic structure collapses to a saturated ferromagnetic one. For *B*||*c* one can also notice additional features, namely a broad maximum and an inflection point of various intensities and eventually, a minimum occurring at high fields. We denote these points as T^*^ and attribute them to enforced alignment of magnetic moments along the applied static magnetic field. These features related to T^*^ are less pronounced for the *B*⊥*c* orientation, for which the antiferromagnetic order is already destroyed by a smaller static magnetic field.

### Heat capacity

**Figure 8:**
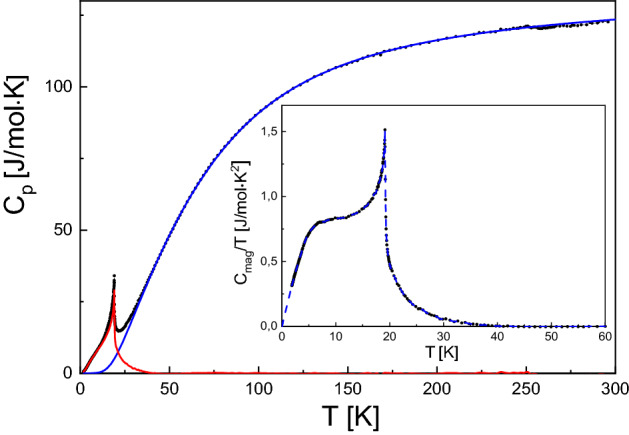
Temperature dependences of the specific heat (*C*_p_). The solid blue curve is a fit by Eq. (2) and the solid red curve is the calculated magnetic contribution (*C*_mag_) to the heat capacity. The inset shows the *C*_mag_/*T* versus *T* plot. The dashed magenta line in the inset is an extrapolation of the data from *T* = 1.85 K to *T* = 0 K and a guide for eyes at higher *T*.

Figure 8 shows the specific heat (*C*_p_) as a function of temperature. A sharp peak appears at about 20 K (maximum value at 19.1 K and the inflection point at 19.2 K), which is the signature of long range AFM ordering of Eu^2+^ moments occurring at this temperature. We assume that sufficiently far above this magnetic peak the ${C}_{p}$ vs $T$ dependence can be approximated by the phonon and electronic (${C}_{el}=\gamma T$) contributions using the following expression^19,20^:

2 $$ C_{p} = C_{ph + el} = \frac{R}{1 - \alpha T}\left[ {9\left( {\frac{T}{{\theta_{D} }}} \right)^{3} \int_{0}^{{\theta_{D} /T}} {\frac{{x^{4} e^{x} }}{{\left( {e^{x} - 1} \right)^{2} }}dx} + \sum\nolimits_{i} {\frac{{m_{i} \left( { \frac{{\theta_{{E_{i} }} }}{T}} \right)^{2} e^{{\theta_{{E_{i} }} /T}} }}{{\left( {e^{{\theta_{{E_{i} }} /T}} - 1} \right)^{2} }}} } \right] + \gamma T, $$

where ${\theta }_{D}$ is a Debye temperature, ${\theta }_{{E}_{i}}$ are Einstein temperatures and ${m}_{i}$ are corresponding multiplicities for each individual optical branch, $\alpha$ stands for an anharmonic coefficient, $\gamma$ is an electronic specific heat coefficient, and $R$ is the gas constant. In order to facilitate analysis, the summation over 12 independent optical branches was grouped into 2 branches with sixfold degeneracy. The *γ* value could not be separately determined from the low-temperature *C*_p_ data because of the presence of *C*_mag_. When allowed *γ* to vary, it tends to correlate strongly with the more significant for goodness of fit, *α* parameter. Therefore, *γ* was fixed at zero for the final fit. The fit to experimental data was performed from 38 to 247 K (far from the AFM peak observed around 20 K) and is indicated by the solid blue curve in Fig. 8. The calculated ${C}_{p}$ at 300 K attains a value of ≈ 123.5 J/(mol K), which is close to the Dulong-Petit high temperature limit ${C}_{V}=3nR=$ 124.7 J/(mol K), where *n* = 5.

The corresponding fit parameters are collected in Table 1. The value of ${\theta }_{D}$ (218 K) obtained is in relatively good agreement with the one obtained from XRD above (261 K). The solid red curve in Fig. 8 shows the calculated magnetic part of the specific heat, *C*_mag_(*T*) = *C*_p_(*T*) − *C*_ph+el_(*T*). The jump of *C*_mag_ at *T*_*N*_ amounts to 28.8 J/(mol K), which is significantly higher than predicted from the mean field theory for a simple antiferromagnetic order (20 J/(mol K))^21^. This behavior is also observed in a similar antiferromagnetically ordered, hexagonal EuMg_2_Bi_2_ and can be attributed to the presence of dynamic correlations, which are not accounted for in the mean field theory^22^.

**Table 1:** The Debye (${\theta }_{D}$), Einstein (${\theta }_{{E}_{i}}$) temperatures and anharmonic coefficient $\left(\alpha \right)$ obtained from the fitting of Eq. (2) to the ${C}_{p}$ vs $T$ dependence.

Parameter	Symbol	Value
Debye temperature	${\theta }_{D}$(K)	217.9 ± 8.2
Einstein temperature	${\theta }_{{E}_{1}}$(K)	119.4 ± 1.9
	${\theta }_{{E}_{2}}$(K)	291.7 ± 1.7
Anharmonic coefficient	$\alpha$(1/K)	(1.037 ± 0.020)10^−4^

The magnetic contribution *S*_mag_(*T*) to the entropy was calculated from the *C*_mag_(*T*) derived from our experiment according to formula (3):

3 $$ S_{mag} \left( T \right) = \int_{0}^{T} {\frac{{C_{mag} }}{T}dT} $$

Due to the lack of experimental data for *C*_mag_(*T*) below 1.85 K, the *C*_mag_/*T* data were extrapolated from 1.85 to 0 K using second order polynomial function (fitted to the low-temperature experimental points). The calculated magnetic entropy between *T* = 0 K and 41 K was determined by numerical integration of the dashed curve, representing *C*_mag_(*T*), shown in the inset in Fig. 8 and is equal to 17.23 J/(mol K). The expected magnetic entropy of one mole of the spin- *J* particles in a magnetic field is given by ${S}_{mag}=R\mathrm{ln}\left(2J+1\right)$. The *J* (for ${S}_{mag}=$ 17.23 J/(mol K)) was estimated to be 3.47—a value well consistent with the presence of Eu^2+^ ions with *J* = 7/2.

**Figure 9:**
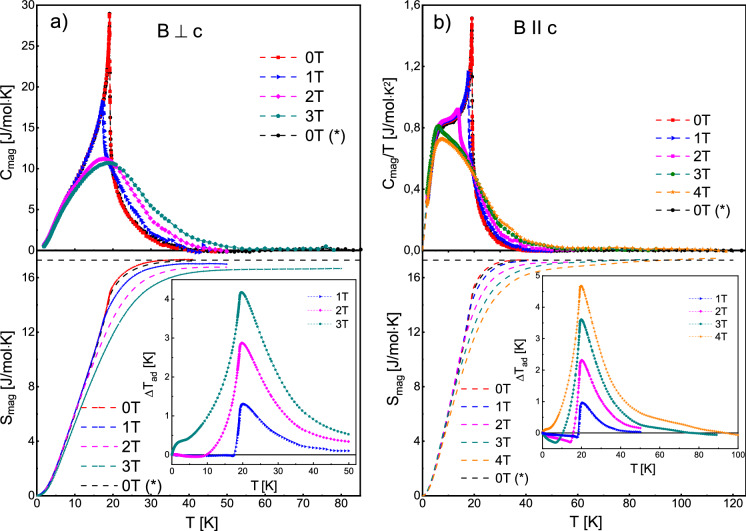
Top panels: temperature dependencies of magnetic specific heat (*C*_mag_) measured at magnetic fields *B*⊥*c* (**a**) and *B*||*c* (**b**) geometries. The solid symbols denote experimental points and the dashed lines are to guide the eye. The bottom panels display the magnetic entropies calculated from corresponding curves in upper panels. Dotted horizontal line indicates infinite temperature limit for the molar magnetic entropy ${S}_{mag}=R\mathrm{ln}\left(2S+1\right)$ for *S* = 7/2. (*) denotes the reference measurement (from the inset in Fig. 8) performed at *B* = 0 T on a larger sample. The insets in (**a**) and (**b**) show adiabatic temperature changes (Δ*T*_ad_) as a function of temperature, obtained from heat capacity data. Figure was created using Origin v. 2019b, https://www.originlab.com/.

For heat capacity measurements at different orientations of the applied magnetic field with respect to crystallographic axes, much smaller (of about 2 mg) bar shape crystal was cut and measured in limited temperature ranges. The magnetic contributions *C*_mag_(*T*) to *C*_p_(*T*) obtained after subtracting the lattice contribution approximated with fitted theoretical curve (Eq. (2)) are shown as *C*_mag_(*T*) dependencies in Fig. 9. At zero external field, the phase transition at 19.1 K could be clearly identified. Upon applying magnetic fields up to 4 T, the peak becomes broader, rounder, shifts to lower temperatures and the transition is monotonously suppressed, similarly to observed in other antiferromagnetically ordered Eu containing compounds^22,23^. Exact positions of the maxima were calculated from the ${C}_{mag}$ versus $T$ dependences for *B*⊥*c* (${T}_{m}^{\perp }$) and *B* || *c* (${T}_{m}^{||}$) geometries and are collected as a function of applied magnetic fields in Table 2. One could conclude from these data that as the magnetic field is increased the jump in the ${C}_{mag}$ progressively decreases and simultaneously the peak position shifts to lower temperature. Clearly stronger field dependence is observed for the *B*⊥*c* geometry, where the jump in the ${C}_{mag}$ completely disappeared at 2 T instead of 3 T (for *B*||*c*), which is consistent with our magnetic susceptibility measurements. The negative peak shift and its disappearance under magnetic field application confirm that there is an antiferromagnetic type order in the compound, which is possibly suppressed at 2 T and 3 T for *B*⊥*c* and *B*||*c* geometries, respectively. As the magnetic field is further increased above 2 T (3 T) the position of the broad peak in the ${C}_{mag}$ vs $T$ dependencies shifts back to higher temperature which is typically observed in ferromagnetic compounds (as larger field further aligns the magnetic moments).

**Table 2:** Temperatures of the anomalies in magnetic specific heat (${C}_{mag}$) calculated from the ${C}_{mag}$ versus $T$ dependences at selected magnetic fields for *B* ⊥ *c* (${T}_{m}^{\perp }$) and *B*||*c* (${T}_{m}^{II}$) geometries.

*B* (T)	$T_{m}^{ \bot }$(K)	${T}_{m}^{II}$(K)
0	19.1	19.1
1	17.3	17.7
2	17.6	13.8
3	18.9	18.1
4	–	18.6

The magnetic entropy *S*_mag_(*T*) was calculated from *C*_mag_(*T*)/*T* dependencies using Eq. (3) (as before, the *C*_mag_/*T* data were fitted to the range between 2.8 and 1.85 K, and extrapolated to 0 K) and the results are shown in Fig. 9 (bottom panels). With increasing magnetic field the magnetic entropy approaches the theoretical limit for *B*⊥*c* and *B*||*c* geometries at increasingly higher temperatures. The non-zero contributions to *C*_mag_ and *S*_mag_ above the ordering temperature likely arise from the dynamic short-range correlations^22^. Some discrepancy is apparent for the *B*⊥*c* geometry (left panel), but it is most probably caused by the narrower measurement temperature ranges and rather falls into the experimental error range (increased by the low sample mass).

Adiabatic temperature change (Δ*T*_ad_) was calculated as^24^:

4 $$ \Delta T_{ad} \left( {T,\Delta B} \right) = T\left( {S,B_{f} } \right) - T\left( {S,B_{i} } \right), $$

where *T*(*S*, *H*) is temperature as a function of the total entropy *S* and magnetic field *B* (initial *B*_*i*_ = 0 and final *B*_*f*_ > 0). The estimated Δ*T*_ad_ from heat capacity data for *B*⊥*c* and *B*||*c* geometries are shown in the insets in Fig. 9. The maximum obtained Δ*T*_ad_ is 4.7 K at 19.8 K and 4 T for *B*||*c* geometry and relatively larger magnetocaloric effect is seen for *B*⊥*c* geometry where the maximum Δ*T*_ad_ is 4.2 K at 19.7 K and 3 T. These values indicate quite high magnetocaloric effect in EuZn_2_As_2_, which is comparable to that in ferromagnetic EuO^25^ or antiferromagnetic EuCu_5_In and EuAg_5_In^26^.

### Mössbauer spectroscopy

**Figure 10:**
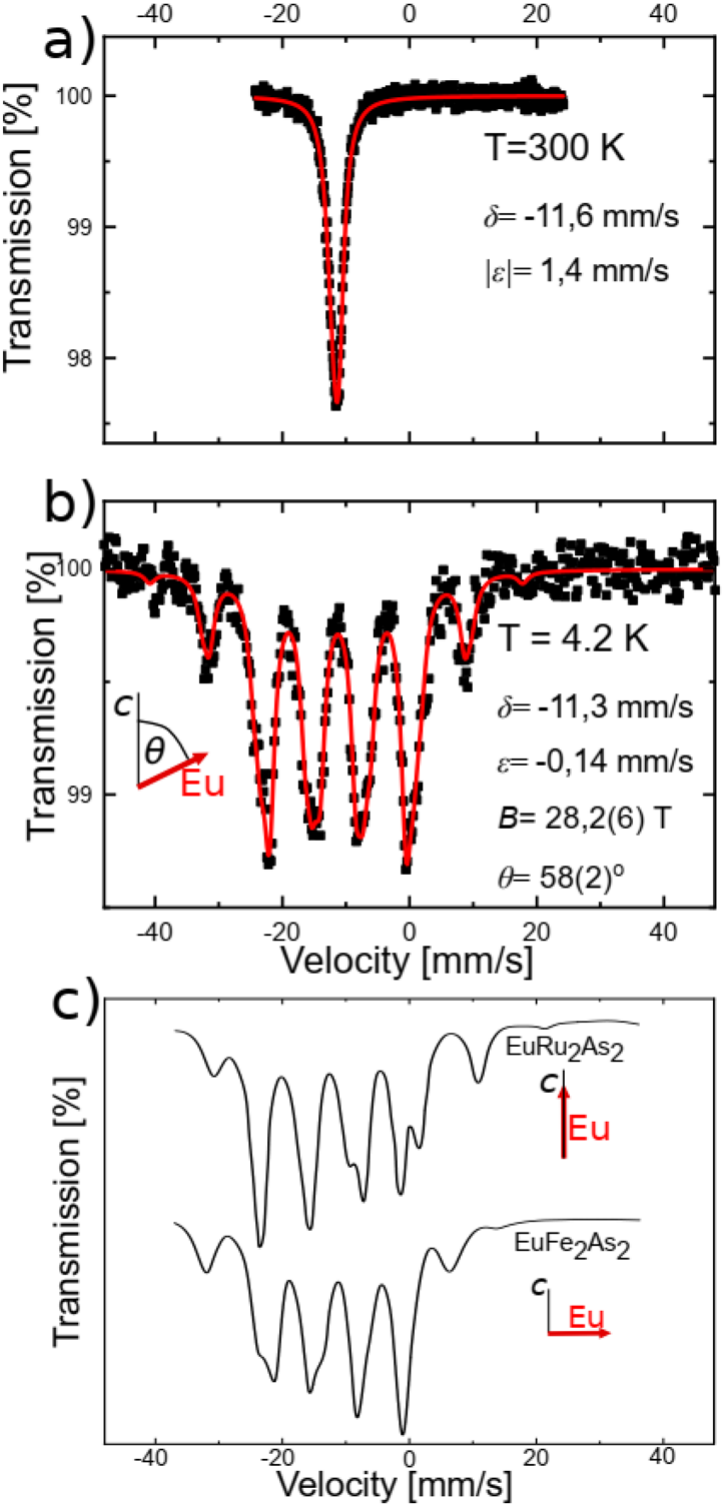
^151^Eu Mössbauer spectra of EuZn_2_As_2_ measured at room temperature and at 4.2 K in panel (**a**) and (**b**), respectively. Experimental data are shown as black squares and the red line is the result of the fit. Panel (**c**) presents spectra for EuRu_2_As_2_ (Eu magnetic moments parallel to the *c*-axis) and EuFe_2_As_2_ (Eu magnetic moments perpendicular to the *c*-axis), after^36^. Figure was created using Origin v. 2019b, https://www.originlab.com/ and Inkscape v. 1.0.1 https://inkscape.org/.

In order to confirm and study in more detail the magnetic structure of EuZn_2_As_2_ deduced above, we used ^151^Eu Mössbauer spectroscopy. This technique is sensitive to the local surrounding of Eu nuclei. The top panel in Fig. 10 presents the ^151^Eu Mössbauer spectrum measured at room temperature. It consists of a single absorption line with the isomer shift, *δ*, of − 11.6 mm/s, which is characteristic for Eu^2+^ ions^27–29^. There is no absorption signal close to 0 mm/s, a value typical for non-magnetic Eu^3+^ ions^30^, indicating a high sample quality (lack of impurities containing Eu^3+^). Since the *c*-axis is a threefold rotation axis (the Eu site has a $\overline{3 }m$ point symmetry, see Fig. 3) one expects that it becomes the principal axis of the electric field gradient tensor ^31^ and the EFG tensor itself is axially symmetric along it. The Eu^2+^ ion has a half-filled 4*f*^7^ electronic configuration with the ^8^*S*_7/2_ ground state, so that the 4*f* electron contribution to the EFG is zero and, thus, the *V*_*zz*_ and the respective quadrupole coupling *ε* are expected to be small, coming from the “lattice” and bonding with adjacent atoms. This is what is observed, at room temperature |*V*_*zz*_| amounts to 35 * 10^20^ V/m^2^, which is smaller than e.g. in EuFeAs_2_ or RbEuFe_4_As_4_ (40 to 50 * 10^20^ V/m^2^) ^32,33^. Larger magnitudes of quadrupole interaction were also reported for similar compounds e.g. EuCd_2_As_2_^34^ or EuFe_2_As_2_^29,35^. It has to be noted, that at room temperature, in the paramagnetic state, Mössbauer spectroscopy is not sensitive to the sign of *V*_*zz*_.

The ^151^Eu spectrum at 4.2 K consists of several groups of peaks, which correspond to the presence of a magnetic field at the Eu nucleus, indicating magnetic order of Eu moments. In the ordered state the expression for the quadrupole coupling constant becomes:

$$\varepsilon = \frac{1}{4}\frac{{ceQ_{g} V_{zz} }}{{E_{\gamma } }}\left( {\frac{{3\cos^{2} \theta - 1}}{2}} \right)$$

In general, it is possible to obtain the information about the angle *θ *between the *c*-axis (principal component axis of the EFG) and the direction of Eu hyperfine field, and, thus, its magnetic moment. However, care should be taken since *V*_*zz*_ and *θ* are fitted simultaneously in the same term and several solutions could provide similar fit quality. In numerous compounds, the quadrupole coupling *ε* at Eu^2+^ has been reported to be negative^29,32–35^. Therefore, in the first attempt, the spectrum was fitted with the *V*_*zz*_ fixed at the value obtained at room temperature but negative, and we obtained *θ* = 58.5 ± 2°. Next, both parameters *V*_*zz*_ and *θ* were allowed to be fitted and *V*_*zz*_ = − 48 * 10^20^ V/m^2^ and *θ* = 58 ± 1° were obtained (fit shown in Fig. 10b). As was mentioned above, recent neutron diffraction study^14^ suggested that Eu moments lie in the plane perpendicular to the *c*-axis and, therefore, we also attempted to fit the spectrum with *θ* fixed at 90°. Such a fit provided a non-physical value of |*V*_*zz*_| six times smaller compared to the value obtained at room temperature, while it is generally observed that the absolute value of *V*_*zz*_ increases as the temperature decreases^32,33,37^, unless there occurs a structural phase transition or strong or uneven change of the lattice parameters with temperature. As apparent from the Fig. 3, the gradient of the electric field could originate mainly from As and Zn ligands. According to XRD data, presented in the Fig. 4, *c/a* ratio changes with temperature by less than 0.1%, while the lattice parameters vary less than 0.4%. Moreover, the As and Zn positional parameters are constant, within the uncertainty level (see Fig. 4c), in the investigated temperature range. The diffraction pattern collected at 14 K shows no sign of possible distortion into a lower symmetry space group. Therefore, we conclude that our Mössbauer spectroscopy results indicate that Eu moments are tilted at angle *θ* = 58 ± 1° with respect to the *c*-axis, i.e. about 32° from the Eu plane, which is almost exactly an angle at which the closest Eu neighbor, As is located (38°), see Fig. 3a.

We note that the obtained value of *ε* = − 0.14 mm/s is smaller than e.g. in EuFe_2_As_2_ (− 1.7 mm/s) ^29^ or EuCd_2_As_2_ (− 0.45 mm/s) ^34^. The magnetic field value *B* = 28.2 T is obtained, which is similar to e.g. EuZn_2_Sb_2_ (28.5 T) ^28^ or EuFe_2_As_2_, EuCo_2_As_2_ (27.4–27.5 T)^29,38^, but somewhat higher than in EuCd_2_As_2_ (25.2 T)^34^ or EuIn_2_As_2_ (26.5 T)^39^. To illustrate the difference in the shape of the ^151^Eu Mössbauer spectrum for different arrangements of Eu magnetic moments in Fig. 10c we show spectra for EuRu_2_As_2_ (Eu magnetic moments parallel to the *c*-axis) and EuFe_2_As_2_ (Eu magnetic moments perpendicular to the *c*-axis)^36^. Canting/tilting of the Eu moments was observed e.g. in EuMn_2_Sb_2_^40^ or EuFe_2_P_2_^41^ and appeared in EuFe_2_As_2_ upon Co or Ni doping^29,42^. Eu moments can couple antiferromagnetically between the Eu planes in a normal fashion, as e.g. in EuFe_2_As_2_^29^, or in more complicated way with a helical order as in EuCo_2_As_2_ or EuIn_2_As_2_^38,39^. However, due to the symmetry of the Eu site, the EFG tensor is axially symmetric about the *c*-axis and our Mössbauer spectroscopy measurements are not sensitive to a rotation of the magnetic moment about the *c*-axis (quadrupole splitting does not vary with horizontal angle change). Therefore, from our Mössbauer measurements it cannot be decisively concluded on a possible type of antiferromagnetic structure.

### Magnetic field and temperature dependent phase diagram

**Figure 11:**
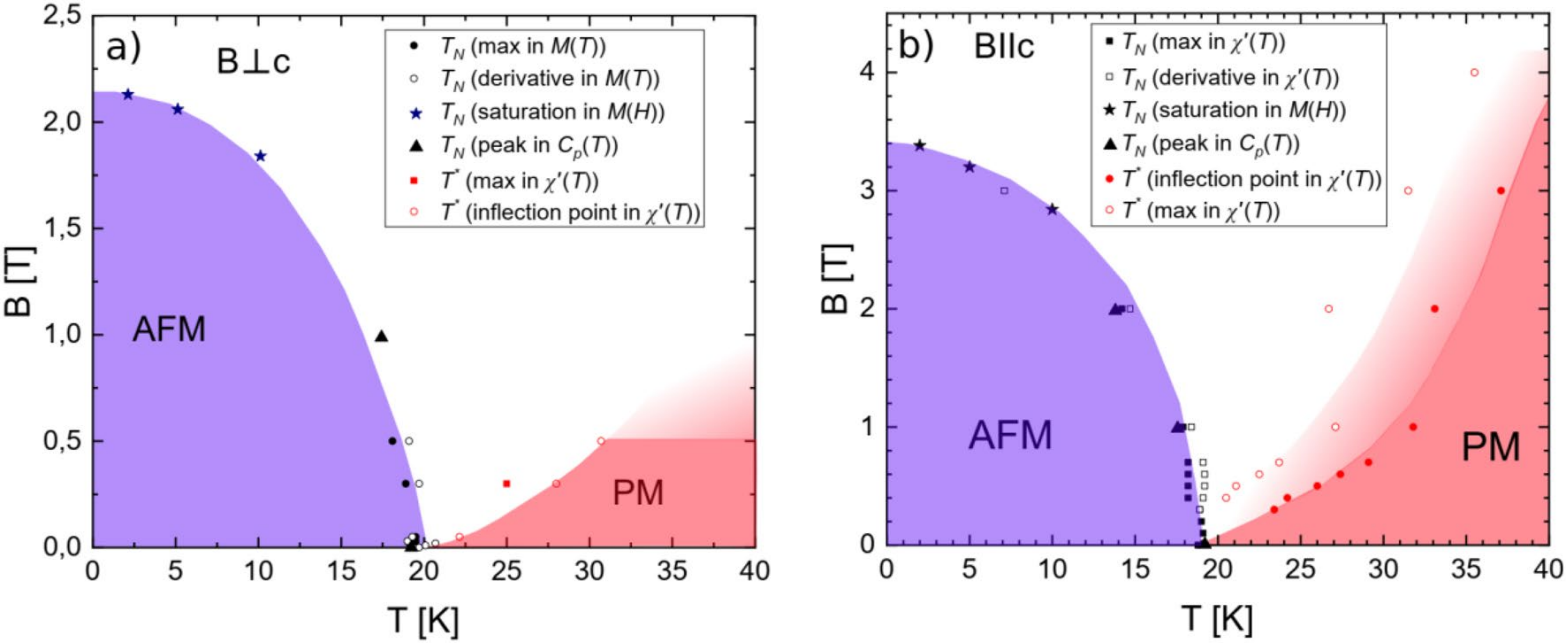
The magnetic field and temperature dependent phase diagram obtained from magnetization, AC magnetic susceptibility and heat capacity measurements showing antiferromagnetic (AFM) and paramagnetic (PM) phase for *B*⊥*c* and *B*||*c* in panel (**a**) and (**b**), respectively. In between the AFM and PM phases magnetic moments are aligned along the direction of the applied magnetic field. Figure was created using Origin v. 2019b, https://www.originlab.com/ and Inkscape v. 1.0.1 https://inkscape.org/.

Figure 11 summarizes results of our studies and presents the magnetic field and temperature dependent phase diagram of EuZn_2_As_2_ obtained from magnetization, AC susceptibility and heat capacity measurements. At zero field it becomes antiferromagnetic at the temperature *T*_*N*_ = 19.2 K. The magnetic ordering temperature decreases with the application of magnetic field at a rate depending on the orientation of the field with respect to the *c*-axis of the crystal. At a sufficiently high magnetic field (of 2.2 T and 3.4 T for *B*⊥*c* and *B*||*c*, respectively) the AFM order is overruled and magnetic moments align along the direction of the applied magnetic field. This type of forced alignment also occurs at lower field but at higher temperatures (i.e. in between the PM and AFM phases at the phase diagram).

## Summary and conclusions

We have successfully grown large (mm size), high quality single crystals of EuZn_2_As_2_ using Sn flux. They were studied by means of X-ray diffraction, electron microscopy, magnetization, AC susceptibility, heat capacity and ^151^Eu Mössbauer spectroscopy. We have found that this compound has a trigonal crystal structure ($P\overline{3 }m1$ space group), and that Eu is in a sole 2+ valence state. Its magnetic moments form a canted antiferromagnetic structure below *T*_*N*_ = 19.2 K with moments tilted from the basal plane as deduced from Mössbauer spectroscopy and from the presence of a ferromagnetic contribution to the magnetization, which has both *c*-axis and the basal plane components.
